# A monoclinic polymorph of chloro­thia­zide

**DOI:** 10.1107/S2056989024006078

**Published:** 2024-06-28

**Authors:** Rowan K. H. Brydson, Alan R. Kennedy

**Affiliations:** a Department of Pure & Applied Chemistry, University of Strathclyde, 295 Cathedral Street, Glasgow G1 1XL, Scotland, United Kingdom; University of Buenos Aires, Argentina

**Keywords:** crystal structure, polymorphism, pharmaceutical

## Abstract

A new polymorph of the diuretic chloro­thia­zide, 6-chloro-1,1-dioxo-2*H*-1,2,4-benzo­thia­zine-7-sulfonamide, C_7_H_6_ClN_3_O_4_S_2_, is described. Crystallized from basic aqueous solution, this monoclinic polymorph is found to be less thermodynamically favoured than the known triclinic polymorph and to feature only N—H⋯O type inter­molecular hydrogen bonds as opposed to the N—H⋯O and N—H⋯N type hydrogen bonds found in the *P*1 form.

## Abstract 2

Accessible from basic aqueous solutions, a new monoclinic polymorph of the diuretic chloro­thia­zide is described.

## 1. Chemical context

Chloro­thia­zide (CTZ) is an Active Pharmaceutical Ingredient (API) used as a diuretic and as an anti­hypertensive drug (Martins *et al.*, 2022 ▸; Steuber *et al.*, 2020 ▸). It has been widely used as a model API in crystallization studies, for instance in screens for solvate and cocrystal forms (Johnston *et al.*, 2011 ▸; Aljohani *et al.*, 2017 ▸; Teng *et al.*, 2020 ▸). Crystal structures of Na and K salt forms have also been reported (Paluch *et al.*, 2010 ▸, 2011 ▸). Finally, CTZ has also been used as a model API in various technique development studies, techniques such as structure solution from powder diffraction (Shankland *et al.*, 1997 ▸), crystal-structure prediction (Johnston *et al.*, 2011 ▸) and high-pressure structural studies of mol­ecular materials (Oswald *et al.*, 2010 ▸). Despite this attention, only one crystalline polymorph of CTZ has been reported as being accessible under ambient conditions. This is Form I CTZ. Indeed Johnston and co-workers screened the crystallization of CTZ from 67 solvents, each under diverse experimental conditions, and only Form I CTZ and solvates of CTZ were identified. Combining these results with a crystal-structure prediction study gave the suggestion that ‘the appearance of an alternative polymorph of CTZ from standard solution crystallizations is unlikely’ (Johnston *et al.*, 2011 ▸).
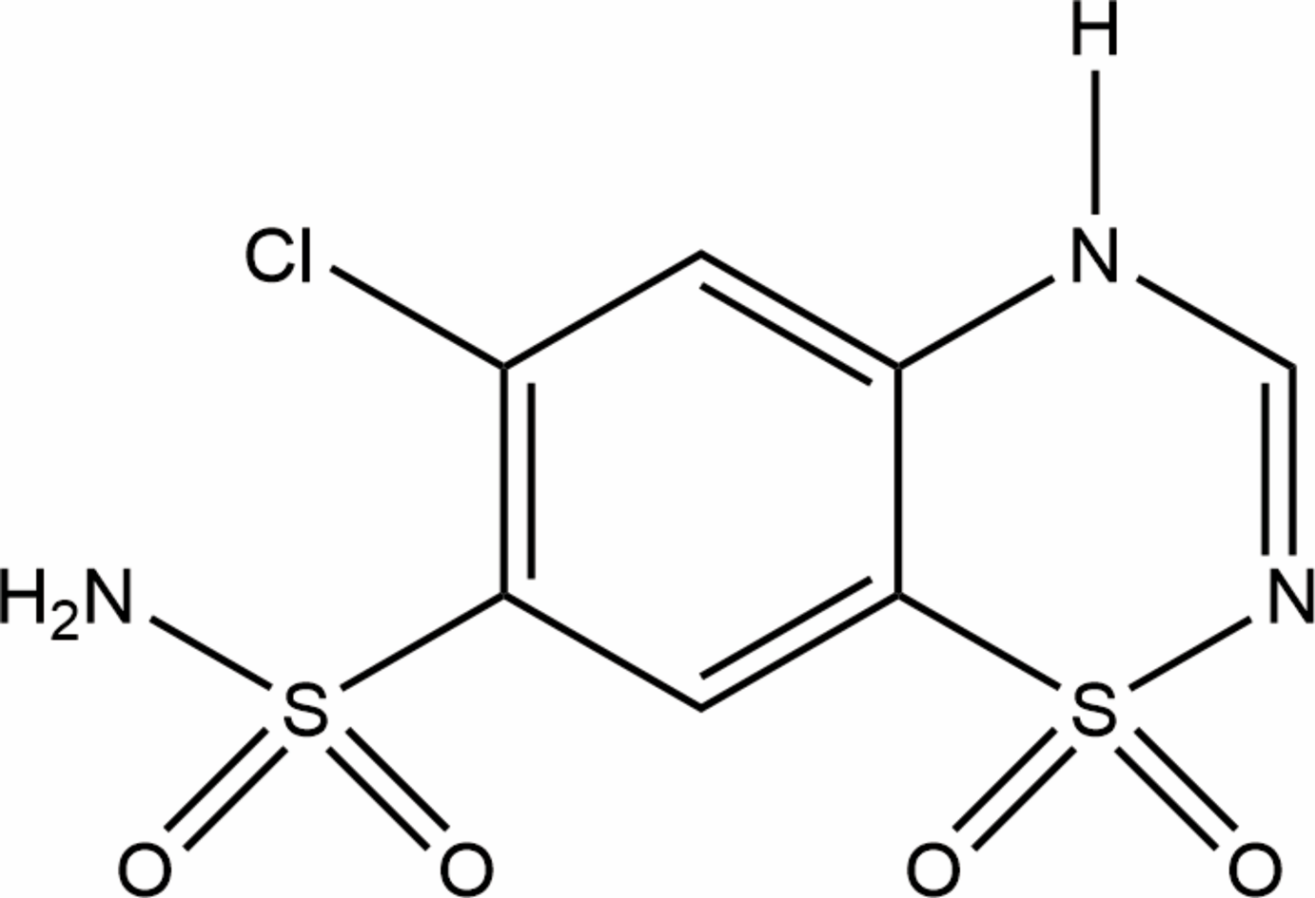


Form I is a triclinic, space group *P*1, structure that was initially reported from a powder diffraction study (Shankland *et al.*, 1997 ▸) with a single-crystal diffraction determination available from Leech *et al.* (2008 ▸). A second closely related polymorph, Form II, was reported in 2010 by Oswald and co-workers ▸. However, this was formed at high pressure (4.4 GPa) by compressing Form I. The transformation is reversible at lower pressures and thus Form II does not persist under standard conditions. Like Form I, Form II is also a triclinic, space group *P*1, structure. Using procedures similar to those outlined by Paluch *et al.* (2010 ▸) to isolate salt forms of CTZ, we crystallized CTZ from water multiple times and often under basic conditions. Despite the earlier predictions, several of these crystallizations produced crystals of a new monoclinic, space group *P*2_1_, form of CTZ suitable for single-crystal X-ray diffraction studies. The structure of this Form III of CTZ is reported herein and is compared to those of its polymorphs.

## 2. Structural commentary

Form III CTZ was originally crystallized from aqueous solution in the presence of Ba(OH)_2_, see the *Synthesis and crystallization* section for details. It was also prepared by similar experiments using either Mg(OH)_2_ or a mix of NaOH and SrCl_2_, in each case the phase was confirmed by X-ray diffraction and by FTIR. See the supporting information for the IR spectra of Forms I and III. The identification of a new polymorph of CTZ is inter­esting as the phase space of this API has been widely studied. For instance, Johnston *et al.* (2011 ▸) performed a crystallization screen using over 400 crystallization procedures and 67 solvents that produced only Form I CTZ and solvate structures. This study showed that, despite CTZ being very sparingly soluble in water (Merk, 1996 ▸), Form I of CTZ could be isolated from aqueous solutions. In our hands, water slurries of CTZ gave only Form I, as shown by IR. The driver for the formation of the new polymorph is thus not using water as the solvent. Relevant known factors that can give polymorphic forms of organic materials are the presence of metal ions (for instance paracetamol, Kennedy *et al.*, 2018 ▸), and change in pH (for instance glycine, Tang *et al.*, 2017 ▸). To test this we repeated the preparation using ammonia rather than Ba(OH)_2_. This metal-ion-free crystallization also produced Form III CTZ, as identified by IR. The driver to Form III generation thus may be the change to higher pH, which here is associated with a much higher aqueous solubility of CTZ.

**Figure 1:**
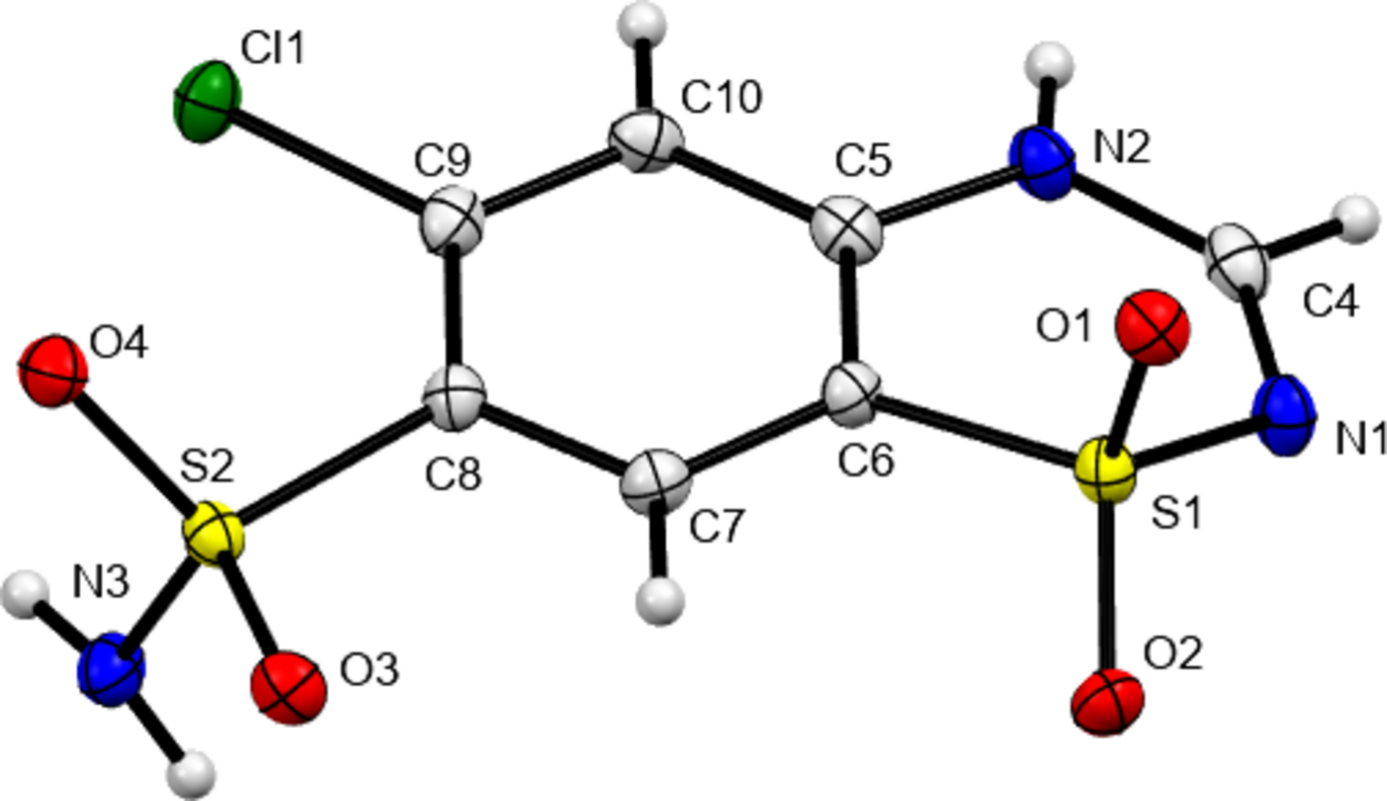
Mol­ecular structure of CTZ Form III with non-H atoms shown as 50% probability ellipsoids. Hydrogen atoms are shown as small spheres of arbitrary size.

The mol­ecular structure of CTZ in Form III is show in Fig. 1 ▸. The secondary amine proton is found bound to N2. This is the commonly described tautomer of CTZ, with the alternate protonation of the sulfonamide nitro­gen (here N1) only described in structures of salt forms (Paluch *et al.*, 2010 ▸, 2011 ▸). The mol­ecular conformation of CTZ in Form III is similar to that found in Form I, with the ring S atom displaced out of the ring plane in the opposite direction to the S—NH_2_ bond vector. However, there are small differences. The magnitude of the C7—C8—S2—N3 torsion angle is 106.7 (3)° in Form III as compared to 109.1° in Form I and the out-of-ring-plane displacement of the S atom is greater in Form III (0.422 *versus* 0.264 Å). Inter­estingly, this latter distortion is similar to that seen in the high-pressure Form II, where the out-of-plane distortion increases with increasing pressure to a maximum of 0.473 Å at 5.9 GPa.

## 3. Supra­molecular features

**Figure 2:**
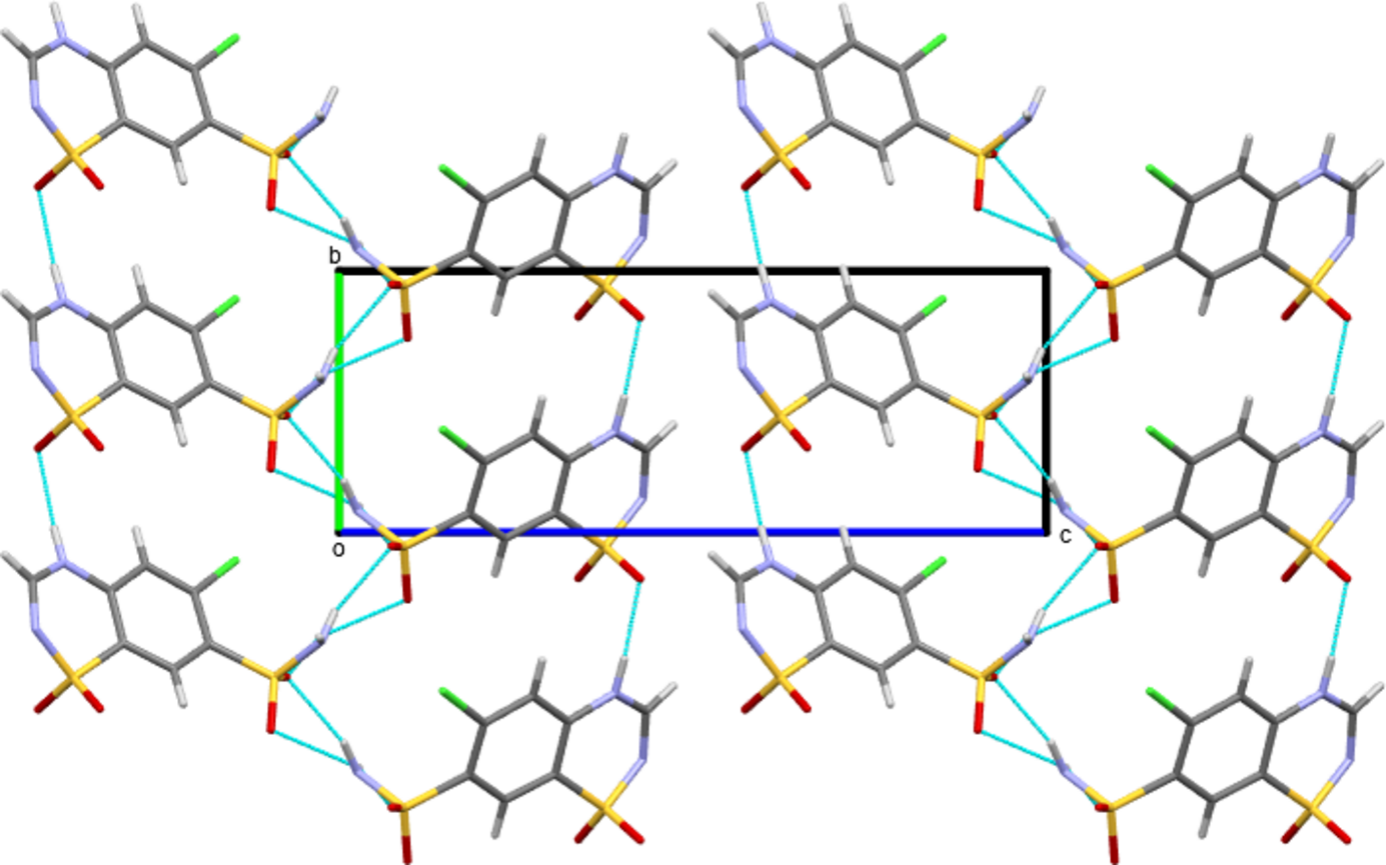
Packing diagram of Form III CTZ with a view along the crystallagraphic *a*-axis direction.

**Figure 3:**
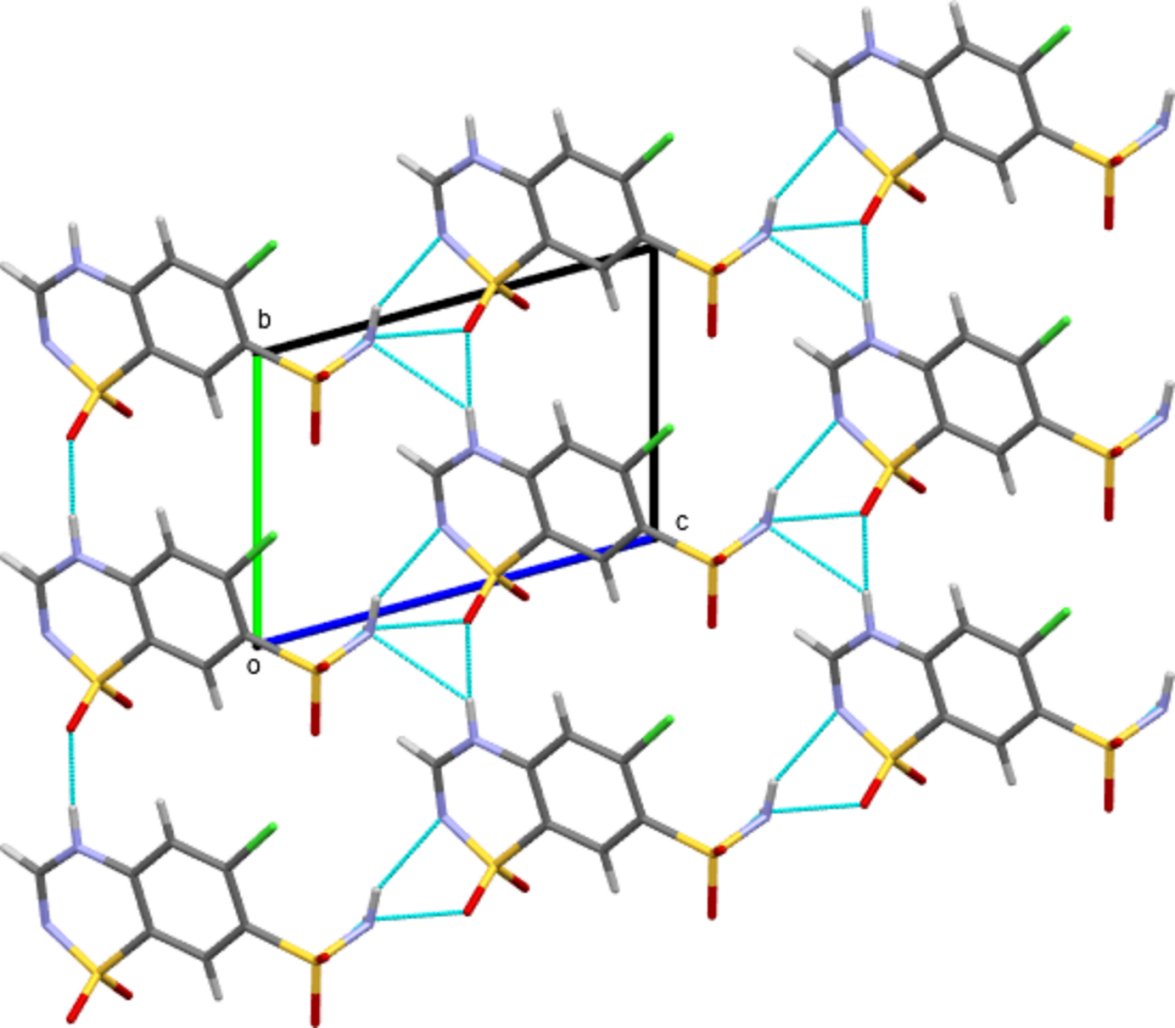
Packing diagram of Form I CTZ with a view along the crystallagraphic *a*-axis direction. Diagram constructed using the CIF file available from Leech *et al.* (2008 ▸).

Form III CTZ forms four independent classical hydrogen bonds all of the N—H⋯O type, see Table 1 ▸. The ring N—H forms a hydrogen bond to an O atom of a neighbouring ring SO_2_ group, whilst the amine H atoms of the SO_2_NH_2_ groups all inter­act with O atoms of neighbouring SO_2_NH_2_ groups. In the case of atom H3*N*, this is a bifurcated inter­action to two mol­ecules. This leaves atom O2 of the ring SO_2_ and all the N atoms unused as hydrogen-bond acceptors. This differs fundamentally from Form I CTZ. There, in addition to three N—H⋯O hydrogen bonds, there are also two N—H⋯N inter­actions. Thus it is an O atom of each of the SO_2_ groups that does not act as an acceptor. The hydrogen bonding of Form III thus consists of only head-to-head and tail-to-tail inter­actions (where the head group is SO_2_NH_2_ and the tail group is the C_3_N_2_S ring), whilst in Form I most of the inter­actions are head-to-tail. The resulting differences in packing can be seen in Figs. 2 ▸ and 3 ▸. In Form III (Fig. 2 ▸), the mix of head-to-head and tail-to-tail inter­actions gives a packing motif with C9—Cl1 vectors pointing both left and right. However, in Form I (Fig. 3 ▸) all equivalent C—Cl vectors are to the right, as would be expected for a space group that features only translational symmetry.

**Figure 4:**
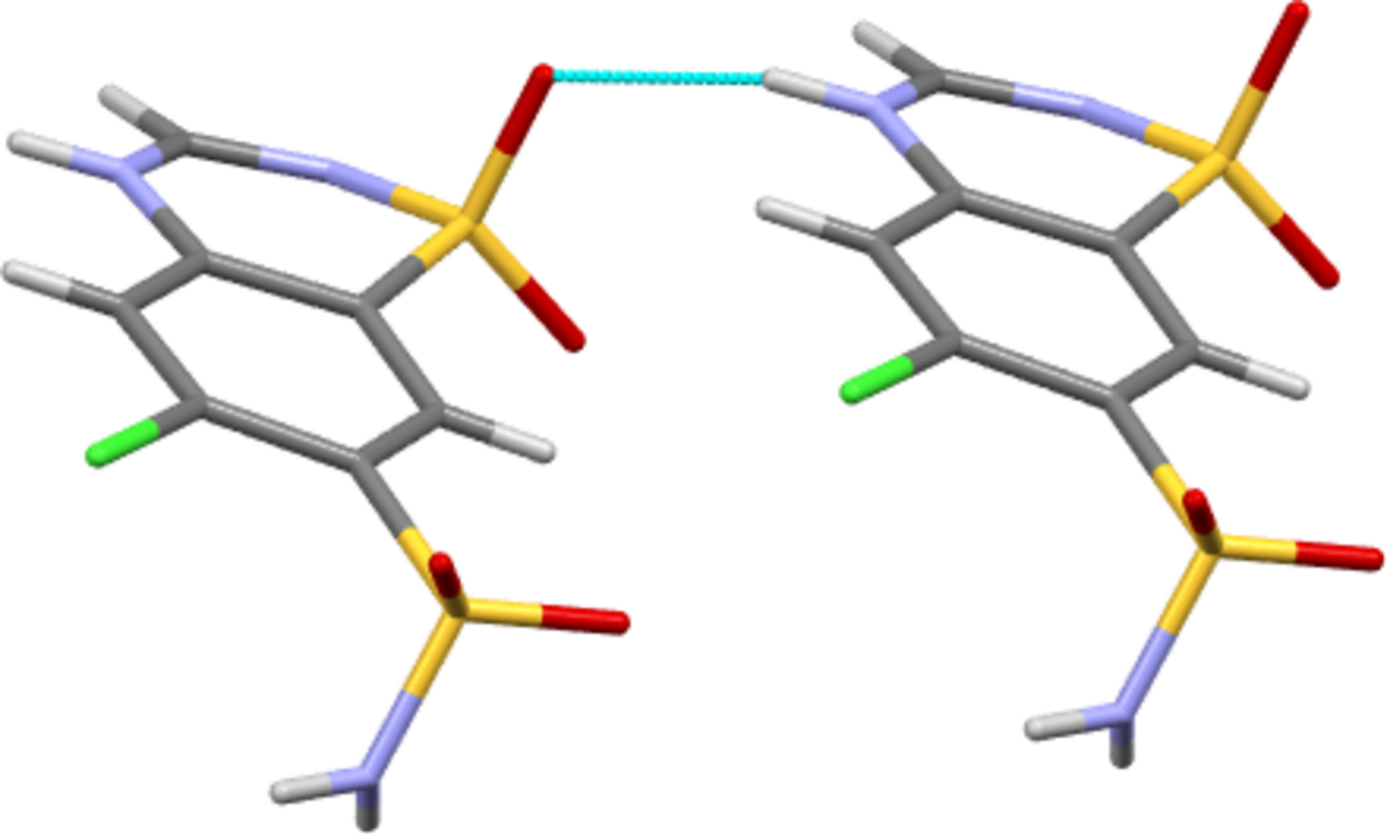
For both polymorphs I and III, this N—H⋯O inter­action type forms the strongest bond between mol­ecular pairs. The motif is shown here for Form III, but a similar motif is also found in Form I.

**Figure 5:**
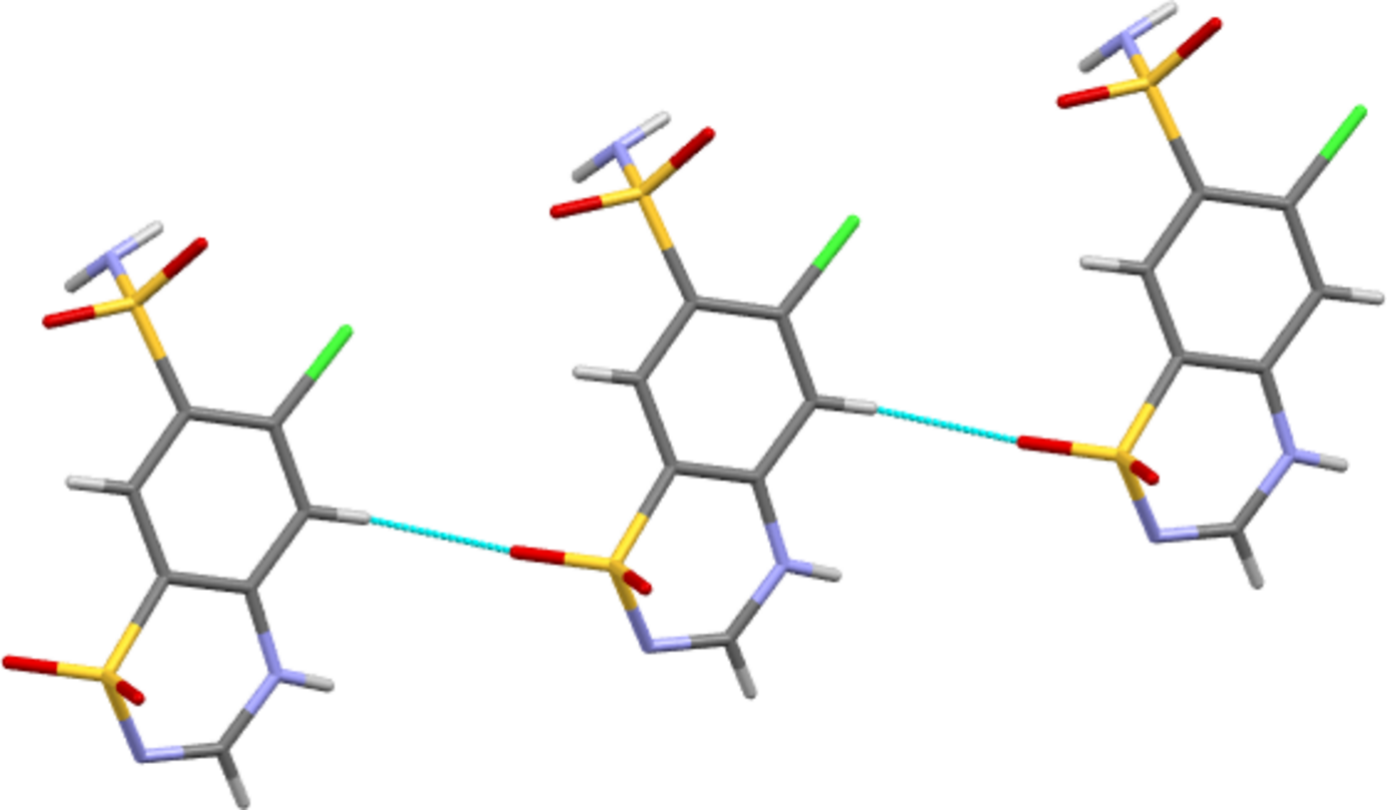
C—H⋯O inter­action motif. Shown here for Form III, but a similar motif is also found in Form I.

**Figure 6:**
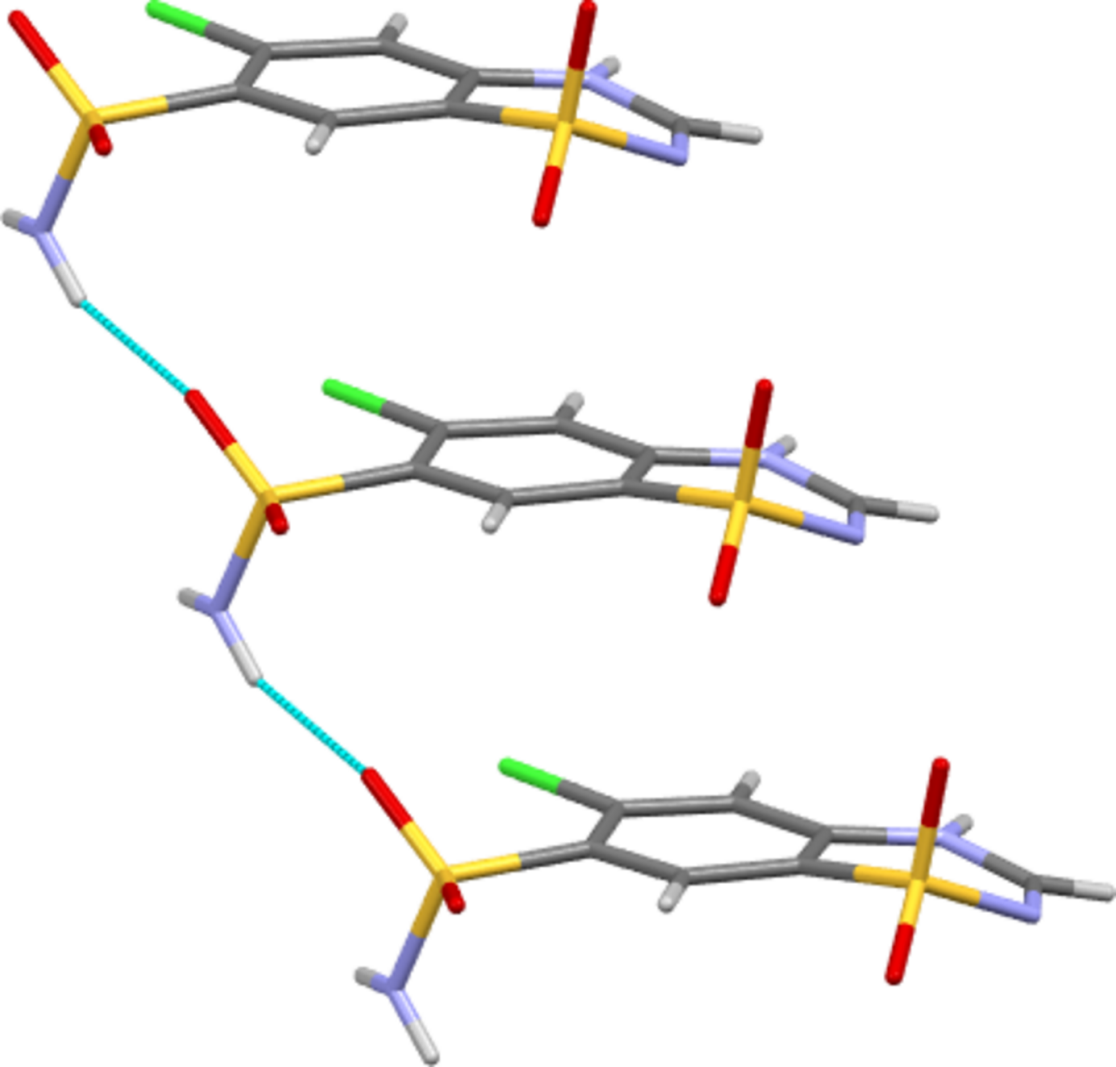
Hydrogen-bond-supported stacking motif corresponding to a translation along the crystallographic *a* axis. Shown here for Form III, but a similar motif is also found in Form I.

The program *CrystalExplorer* (Spackman *et al.*, 2021 ▸) was used to investigate the inter­molecular inter­actions of Forms I and III. Hirshfeld surfaces, fingerprint plots and inter­action energy details are given in the supporting information. The three strongest inter­molecular inter­action types were found to be common to both polymorphs. In each case, the strongest pair inter­action was that based around the ring-to-ring N—H⋯O hydrogen bond shown in Fig. 4 ▸. This had an energy of −58.5 kJ mol^−1^ for Form III and −41.1 kJ mol^−1^ for Form I. Perhaps surprisingly, for each polymorph it is the non-classical C—H⋯O hydrogen-bond inter­action shown in Fig. 5 ▸ that is the next strongest, with energy values of −30.4 and −40.8 kJ mol^−1^ for Forms III and I, respectively. The third common inter­molecular motif is the hydrogen-bond-supported stack motif, shown in Fig. 6 ▸, that corresponds to translation along the crystallographic *a* axis. This has energy values of −24.1 and −28.6 kJ mol^−1^ for Forms III and I, respectively.

Totalling all the pairwise inter­action energies gives −296.0 kJ mol^−1^ for Form III and −308.8 kJ mol^−1^ for Form I. This suggests that triclinic Form I is thermodynamically favoured over the newly described monoclinic Form III. This assignment is supported by both the melting point data (see *Synthesis and crystallization*) and by slurry experiments. After 10 days with cyclic heating to 350 K, a sample of Form III partially dissolved in water had transformed to Form I as shown by FTIR. A similar experiment using Form I material gave no transformation.

## 4. Database survey

A search of the Cambridge Structural Database (CSD, version 5.45 updates to March 2024; Groom *et al.*, 2016 ▸) found two polymorphic forms of CTZ, the ambient condition Form I (Leech *et al.*, 2008 ▸) and the high-pressure only Form II (Oswald *et al.*, 2010 ▸). The structures of many solvate and cocrystal forms of CTZ have also been reported (for examples, see: Johnston *et al.*, 2011 ▸; Aljohani *et al.*, 2017 ▸; Teng *et al.*, 2020 ▸). Johnston *et al.* also list approximately 135 predicted crystal structures of CTZ that have lattice energies that lie within 15 kJ mol^−1^ of their global minimum. Of these, the unit cell of af74 is perhaps closest to that found for Form III (predicted *P*2_1_, *a*, *b*, *c* = 4.9301, 6.7048, 17.268 Å, β = 93.694°). This structure had a predicted lattice energy that was approximately 8.7 kJ mol^−1^ less stable than that of their predicted Form I structure.

## 5. Synthesis and crystallization

CTZ Form I was purchased from Thermo Scientific. 0.1 g (3.4 mmol) of Form I CTZ formed a slurry with 15 cm^3^ of deionized water. An equimolar amount of Ba(OH)_2_·8H_2_O was added and the slurry clarified to a solution. After filtering, this solution was left to evaporate for 5 days after which point colourless crystals of Form III CTZ had formed. Similar experiments using Mg(OH)_2_ or a mix of NaOH and SrCl_2_ in place of Ba(OH)_2_ also gave crystals of Form III CTZ. A final slurry of 0.1 g of Form I CTZ in 15 cm^3^ of deionized water had 35% aqueous ammonia solution added to it dropwise until the solution clarified. After evaporation for 7 days, a white powder formed that was shown to be Form III CTZ by FTIR.

FTIR measurements utilized an Agilent Technologies ATR-FTIR spectrometer. Form I CTZ; FTIR (cm^−1^) 3344, 3257, 3081, 1508, 1310, 1167, 953, 515, m.p. 615–616 K dec. (lit. 616–616.5 K dec.; Merk, 1996 ▸). Form III CTZ; FTIR (cm^−1^) 3697, 3421, 3307, 3006, 1572, 1299, 1093, 893, 674, 500, m.p. 548–549 K dec.

## 6. Refinement

Crystal data, data collection and structure refinement details are summarized in Table 2 ▸. H atoms bound to C atoms were placed in expected geometric positions and treated in riding modes with C—H = 0.95 Å and with *U*_iso_(H) = 1.2*U*_eq_(C). H atoms bound to N were refined isotropically with N—H restrained to 0.88 (1) Å. The structure was refined as a two-component inversion twin.

## Supplementary Material

Crystal structure: contains datablock(s) I, global. DOI: 10.1107/S2056989024006078/vu2004sup1.cif

Structure factors: contains datablock(s) I. DOI: 10.1107/S2056989024006078/vu2004Isup2.hkl

FTIR spectra and Crystal Explorer calculations. DOI: 10.1107/S2056989024006078/vu2004sup3.docx

CCDC reference: 2364581

Additional supporting information:  crystallographic information; 3D view; checkCIF report

## Figures and Tables

**Table 1:** Hydrogen-bond geometry (Å, °)

*D*—H⋯*A*	*D*—H	H⋯*A*	*D*⋯*A*	*D*—H⋯*A*
N2—H1*N*⋯O1^i^	0.88 (1)	2.00 (2)	2.815 (5)	154 (4)
N3—H2*N*⋯O4^ii^	0.88 (1)	2.18 (2)	3.015 (4)	158 (4)
N3—H3*N*⋯O4^iii^	0.87 (1)	2.22 (4)	2.963 (4)	143 (5)
N3—H3*N*⋯O3^iv^	0.87 (1)	2.46 (5)	2.870 (4)	109 (4)

Symmetry codes: (i) 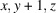; (ii) 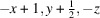; (iii) 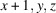; (iv) 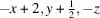.

**Table 2:** Experimental details

Crystal data	
Chemical formula	C_7_H_6_ClN_3_O_4_S_2_
*M*_r_	295.72
Crystal system, space group	Monoclinic, *P*2_1_
Temperature (K)	100
*a*, *b*, *c* (Å)	4.8296 (1), 6.2703 (1), 16.9551 (2)
β (°)	92.214 (1)
*V* (Å^3^)	513.07 (2)
*Z*	2
Radiation type	Cu *K*α
μ (mm^−1^)	7.23
Crystal size (mm)	0.23 × 0.12 × 0.05

Data collection	
Diffractometer	Rigaku Synergy-i
Absorption correction	Multi-scan (*CrysAlis PRO*; Rigaku OD, 2023 ▸)
*T*_min_, *T*_max_	0.393, 1.000
No. of measured, independent and observed [*I* > 2σ(*I*)] reflections	8271, 1953, 1895
*R*_int_	0.046
(sin θ/λ)_max_ (Å^−1^)	0.615

Refinement	
*R*[*F*^2^ > 2σ(*F*^2^)], *wR*(*F*^2^), *S*	0.028, 0.078, 1.06
No. of reflections	1953
No. of parameters	167
No. of restraints	4
H-atom treatment	H atoms treated by a mixture of independent and constrained refinement
Δρ_max_, Δρ_min_ (e Å^−3^)	0.28, −0.33
Absolute structure	Refined as an inversion twin.
Absolute structure parameter	0.00 (2)

Computer programs: *CrysAlis PRO* (Rigaku OD, 2023 ▸), *SHELXT* (Sheldrick, 2015*a* ▸), *SHELXL2018* (Sheldrick, 2015*b* ▸), *Mercury* (Macrae *et al.*, 2020 ▸) and *WinGX* (Farrugia, 2012 ▸).

## supplementary crystallographic information

### 6-Chloro-1,1-dioxo-2H-1,2,4-benzothiazine-7-sulfonamide Crystal data

**Table:**

C_7_H_6_ClN_3_O_4_S_2_	*F*(000) = 300
*M**_r_* = 295.72	*D*_x_ = 1.914 Mg m^−^^3^
Monoclinic, *P*2_1_	Cu *K*α radiation, λ = 1.54184 Å
*a* = 4.8296 (1) Å	Cell parameters from 6651 reflections
*b* = 6.2703 (1) Å	θ = 2.6–71.3°
*c* = 16.9551 (2) Å	µ = 7.23 mm^−^^1^
β = 92.214 (1)°	*T* = 100 K
*V* = 513.07 (2) Å^3^	Tablet, colourless
*Z* = 2	0.23 × 0.12 × 0.05 mm

### 6-Chloro-1,1-dioxo-2H-1,2,4-benzothiazine-7-sulfonamide Data collection

**Table:**

Rigaku Synergy-i diffractometer	1895 reflections with *I* > 2σ(*I*)
Radiation source: microsource tube	*R*_int_ = 0.046
ω scans	θ_max_ = 71.4°, θ_min_ = 2.6°
Absorption correction: multi-scan (CrysAlisPro; Rigaku OD, 2023)	*h* = −5→5
*T*_min_ = 0.393, *T*_max_ = 1.000	*k* = −7→7
8271 measured reflections	*l* = −20→20
1953 independent reflections	

### 6-Chloro-1,1-dioxo-2H-1,2,4-benzothiazine-7-sulfonamide Refinement

**Table:**

Refinement on *F*^2^	Hydrogen site location: mixed
Least-squares matrix: full	H atoms treated by a mixture of independent and constrained refinement
*R*[*F*^2^ > 2σ(*F*^2^)] = 0.028	*w* = 1/[σ^2^(*F*_o_^2^) + (0.0518*P*)^2^ + 0.0232*P*] where *P* = (*F*_o_^2^ + 2*F*_c_^2^)/3
*wR*(*F*^2^) = 0.078	(Δ/σ)_max_ < 0.001
*S* = 1.06	Δρ_max_ = 0.28 e Å^−^^3^
1953 reflections	Δρ_min_ = −0.33 e Å^−^^3^
167 parameters	Absolute structure: Refined as an inversion twin.
4 restraints	Absolute structure parameter: 0.00 (2)

### 6-Chloro-1,1-dioxo-2H-1,2,4-benzothiazine-7-sulfonamide Special details

**Table:**

Geometry. All esds (except the esd in the dihedral angle between two l.s. planes) are estimated using the full covariance matrix. The cell esds are taken into account individually in the estimation of esds in distances, angles and torsion angles; correlations between esds in cell parameters are only used when they are defined by crystal symmetry. An approximate (isotropic) treatment of cell esds is used for estimating esds involving l.s. planes.
Refinement. Refined as a 2-component inversion twin.

### 6-Chloro-1,1-dioxo-2H-1,2,4-benzothiazine-7-sulfonamide Fractional atomic coordinates and isotropic or equivalent isotropic displacement parameters (Å^2^)

**Table:**

	*x*	*y*	*z*	*U*_iso_*/*U*_eq_	
S1	1.24147 (16)	−0.05644 (13)	0.37597 (4)	0.0160 (2)	
S2	0.69084 (16)	−0.05479 (14)	0.09149 (4)	0.0160 (2)	
Cl1	0.39002 (17)	0.39240 (15)	0.14683 (5)	0.0208 (2)	
O1	1.0629 (5)	−0.1778 (5)	0.42573 (14)	0.0197 (6)	
O2	1.4525 (5)	−0.1751 (5)	0.33834 (15)	0.0208 (6)	
O3	0.8308 (5)	−0.2558 (4)	0.09669 (15)	0.0190 (6)	
O4	0.3972 (5)	−0.0527 (5)	0.07447 (14)	0.0206 (6)	
N1	1.3822 (7)	0.1311 (6)	0.42965 (18)	0.0196 (7)	
N2	1.0378 (7)	0.3842 (6)	0.38987 (18)	0.0189 (7)	
N3	0.8293 (6)	0.0843 (6)	0.02524 (17)	0.0198 (7)	
C4	1.2577 (8)	0.3159 (7)	0.4339 (2)	0.0197 (8)	
H4	1.331004	0.412655	0.472437	0.024*	
C5	0.9319 (8)	0.2767 (6)	0.3236 (2)	0.0168 (7)	
C6	1.0335 (7)	0.0753 (7)	0.3054 (2)	0.0152 (7)	
C7	0.9550 (7)	−0.0232 (7)	0.23446 (19)	0.0162 (8)	
H7	1.034802	−0.155951	0.220703	0.019*	
C8	0.7598 (7)	0.0731 (6)	0.1838 (2)	0.0153 (7)	
C9	0.6444 (7)	0.2702 (6)	0.2052 (2)	0.0178 (8)	
C10	0.7320 (7)	0.3739 (7)	0.2740 (2)	0.0176 (8)	
H10	0.657180	0.508943	0.287066	0.021*	
H1N	0.989 (9)	0.514 (4)	0.403 (3)	0.020 (12)*	
H2N	0.731 (8)	0.193 (5)	0.007 (3)	0.022 (12)*	
H3N	1.009 (3)	0.099 (10)	0.028 (3)	0.037 (14)*	

### 6-Chloro-1,1-dioxo-2H-1,2,4-benzothiazine-7-sulfonamide Atomic displacement parameters (Å^2^)

**Table:**

	*U*^11^	*U*^22^	*U*^33^	*U*^12^	*U*^13^	*U*^23^
S1	0.0188 (4)	0.0138 (5)	0.0153 (4)	0.0004 (4)	−0.0016 (3)	−0.0004 (3)
S2	0.0170 (4)	0.0166 (5)	0.0143 (4)	−0.0015 (4)	−0.0002 (3)	−0.0013 (3)
Cl1	0.0216 (4)	0.0179 (5)	0.0227 (4)	0.0014 (4)	−0.0037 (3)	0.0033 (3)
O1	0.0249 (14)	0.0153 (15)	0.0190 (13)	0.0007 (11)	0.0014 (11)	0.0012 (10)
O2	0.0192 (12)	0.0213 (16)	0.0219 (13)	0.0056 (11)	−0.0009 (10)	−0.0013 (11)
O3	0.0230 (13)	0.0131 (15)	0.0209 (13)	−0.0012 (11)	0.0009 (10)	−0.0020 (10)
O4	0.0180 (12)	0.0241 (16)	0.0198 (11)	−0.0021 (12)	0.0000 (9)	−0.0036 (12)
N1	0.0239 (16)	0.0163 (18)	0.0182 (14)	−0.0016 (14)	−0.0042 (12)	−0.0027 (12)
N2	0.0235 (16)	0.0160 (19)	0.0170 (15)	−0.0006 (14)	−0.0005 (12)	−0.0031 (12)
N3	0.0191 (16)	0.0214 (18)	0.0188 (15)	0.0004 (14)	0.0004 (12)	0.0042 (14)
C4	0.0234 (19)	0.021 (2)	0.0146 (17)	−0.0052 (16)	0.0014 (14)	0.0008 (16)
C5	0.0198 (18)	0.0141 (19)	0.0166 (16)	−0.0031 (15)	0.0029 (13)	0.0003 (15)
C6	0.0167 (17)	0.0138 (19)	0.0151 (15)	0.0003 (15)	0.0004 (13)	0.0015 (14)
C7	0.0172 (16)	0.013 (2)	0.0187 (16)	0.0003 (15)	0.0012 (12)	0.0010 (14)
C8	0.0156 (16)	0.0151 (19)	0.0151 (15)	−0.0020 (14)	−0.0002 (13)	0.0008 (14)
C9	0.0170 (18)	0.018 (2)	0.0179 (16)	−0.0011 (16)	0.0004 (14)	0.0046 (15)
C10	0.0203 (17)	0.014 (2)	0.0187 (17)	0.0018 (16)	0.0031 (13)	0.0006 (14)

### 6-Chloro-1,1-dioxo-2H-1,2,4-benzothiazine-7-sulfonamide Geometric parameters (Å, º)

**Table:**

S1—O2	1.432 (3)	N2—H1N	0.875 (14)
S1—O1	1.446 (3)	N3—H2N	0.880 (14)
S1—N1	1.621 (3)	N3—H3N	0.872 (14)
S1—C6	1.740 (4)	C4—H4	0.9500
S2—O3	1.431 (3)	C5—C6	1.394 (6)
S2—O4	1.436 (2)	C5—C10	1.396 (5)
S2—N3	1.590 (3)	C6—C7	1.392 (5)
S2—C8	1.779 (4)	C7—C8	1.388 (5)
Cl1—C9	1.727 (4)	C7—H7	0.9500
N1—C4	1.308 (5)	C8—C9	1.409 (5)
N2—C4	1.345 (5)	C9—C10	1.387 (5)
N2—C5	1.391 (5)	C10—H10	0.9500
			
O2—S1—O1	115.93 (18)	N1—C4—H4	116.3
O2—S1—N1	109.70 (17)	N2—C4—H4	116.3
O1—S1—N1	107.51 (16)	N2—C5—C6	119.7 (3)
O2—S1—C6	110.02 (16)	N2—C5—C10	120.0 (4)
O1—S1—C6	108.02 (16)	C6—C5—C10	120.3 (3)
N1—S1—C6	105.06 (19)	C7—C6—C5	120.4 (3)
O3—S2—O4	118.73 (17)	C7—C6—S1	120.9 (3)
O3—S2—N3	108.43 (17)	C5—C6—S1	118.5 (3)
O4—S2—N3	106.97 (16)	C8—C7—C6	119.8 (4)
O3—S2—C8	105.71 (17)	C8—C7—H7	120.1
O4—S2—C8	108.81 (16)	C6—C7—H7	120.1
N3—S2—C8	107.76 (18)	C7—C8—C9	119.3 (3)
C4—N1—S1	119.3 (3)	C7—C8—S2	116.6 (3)
C4—N2—C5	123.5 (4)	C9—C8—S2	124.0 (3)
C4—N2—H1N	112 (3)	C10—C9—C8	121.1 (3)
C5—N2—H1N	124 (3)	C10—C9—Cl1	117.4 (3)
S2—N3—H2N	116 (3)	C8—C9—Cl1	121.4 (3)
S2—N3—H3N	117 (3)	C9—C10—C5	118.9 (4)
H2N—N3—H3N	118 (5)	C9—C10—H10	120.5
N1—C4—N2	127.3 (4)	C5—C10—H10	120.5
			
O2—S1—N1—C4	143.1 (3)	S1—C6—C7—C8	171.4 (3)
O1—S1—N1—C4	−90.0 (3)	C6—C7—C8—C9	0.2 (5)
C6—S1—N1—C4	24.8 (3)	C6—C7—C8—S2	175.6 (3)
S1—N1—C4—N2	−9.9 (5)	O3—S2—C8—C7	9.1 (3)
C5—N2—C4—N1	−10.0 (6)	O4—S2—C8—C7	137.6 (3)
C4—N2—C5—C6	7.7 (5)	N3—S2—C8—C7	−106.7 (3)
C4—N2—C5—C10	−169.9 (3)	O3—S2—C8—C9	−175.7 (3)
N2—C5—C6—C7	−172.1 (3)	O4—S2—C8—C9	−47.1 (4)
C10—C5—C6—C7	5.5 (5)	N3—S2—C8—C9	68.5 (3)
N2—C5—C6—S1	12.0 (5)	C7—C8—C9—C10	3.0 (5)
C10—C5—C6—S1	−170.4 (3)	S2—C8—C9—C10	−172.1 (3)
O2—S1—C6—C7	40.0 (4)	C7—C8—C9—Cl1	−177.2 (3)
O1—S1—C6—C7	−87.4 (3)	S2—C8—C9—Cl1	7.7 (5)
N1—S1—C6—C7	158.0 (3)	C8—C9—C10—C5	−2.0 (5)
O2—S1—C6—C5	−144.1 (3)	Cl1—C9—C10—C5	178.2 (3)
O1—S1—C6—C5	88.4 (3)	N2—C5—C10—C9	175.3 (3)
N1—S1—C6—C5	−26.1 (3)	C6—C5—C10—C9	−2.3 (5)
C5—C6—C7—C8	−4.4 (5)		

### 6-Chloro-1,1-dioxo-2H-1,2,4-benzothiazine-7-sulfonamide Hydrogen-bond geometry (Å, º)

**Table:**

*D*—H···*A*	*D*—H	H···*A*	*D*···*A*	*D*—H···*A*
N2—H1*N*···O1^i^	0.88 (1)	2.00 (2)	2.815 (5)	154 (4)
N3—H2*N*···O4^ii^	0.88 (1)	2.18 (2)	3.015 (4)	158 (4)
N3—H3*N*···O4^iii^	0.87 (1)	2.22 (4)	2.963 (4)	143 (5)
N3—H3*N*···O3^iv^	0.87 (1)	2.46 (5)	2.870 (4)	109 (4)

Symmetry codes: (i) *x*, *y*+1, *z*; (ii) −*x*+1, *y*+1/2, −*z*; (iii) *x*+1, *y*, *z*; (iv) −*x*+2, *y*+1/2, −*z*.
