# Supplementary material for: A monoclinic polymorph of chloro­thia­zide
Source: Acta Crystallogr E Crystallogr Commun. 2024 Jun 28;80(Pt 7):806–10. doi: 10.1107/S2056989024006078 (PMC11223696; doi:10.1107/S2056989024006078)
Supplement: Supplementary file 3 [file e-80-00806-sup3.docx]

Supplementary information for

“A monoclinic polymorph of chlorothiazide”

by Rowan K. H. Brydson & Alan R. Kennedy.

1. **Infra-red.**
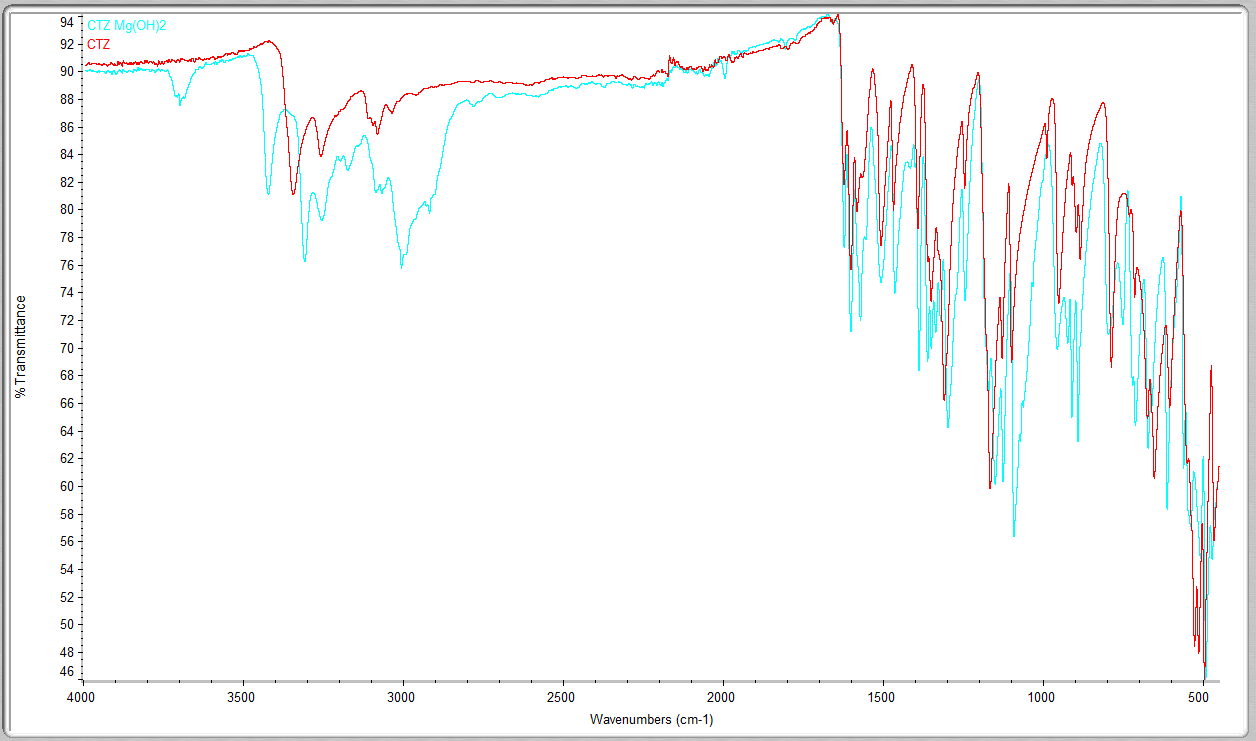


Figure 1. Blue = monoclinic Form III spectrum. Red = triclinic Form I spectrum.

1. **Hirshfeld Surfaces**


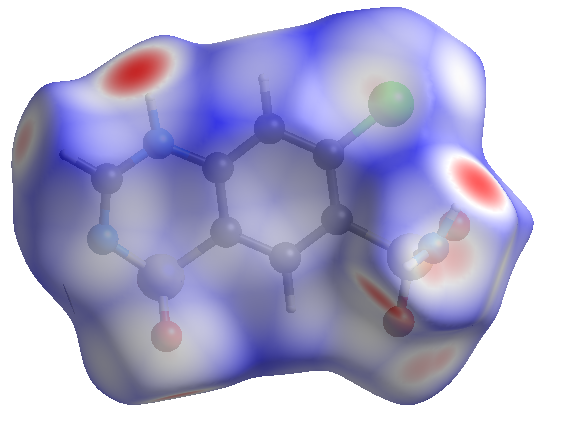

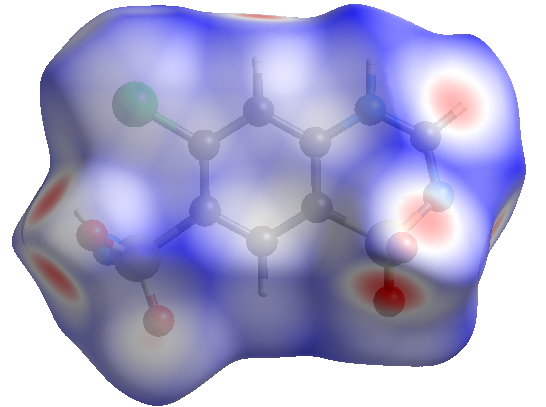


Figure 2. Monoclinic Form III d_norm_ surface.


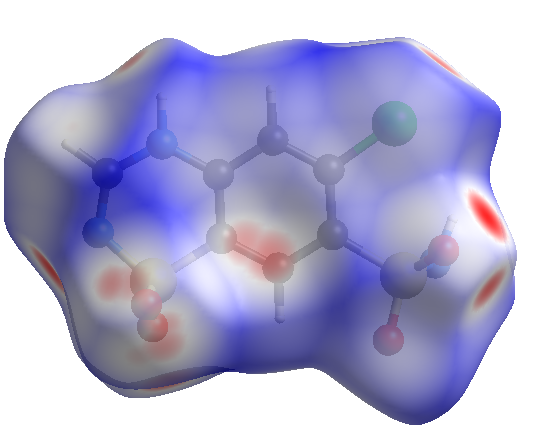

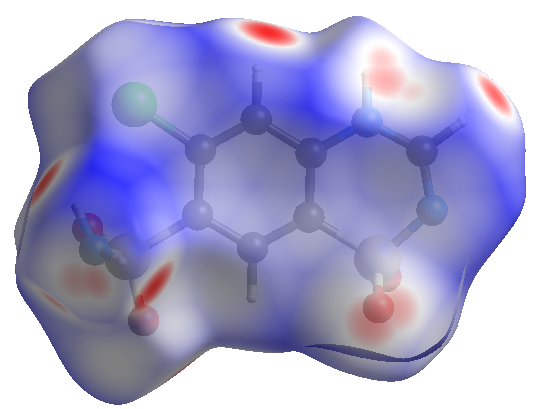


Figure 3. Triclinic Form I d_norm_ surface.


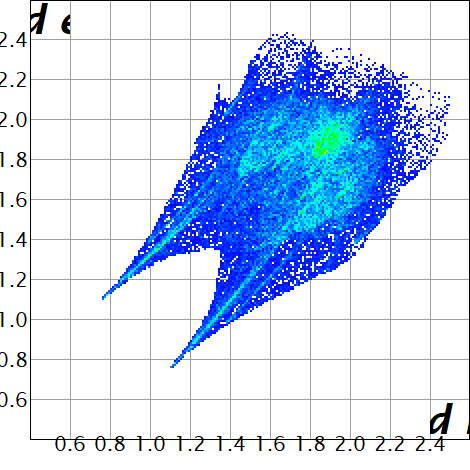


Figure 4. Monoclinic Form III fingerprint.


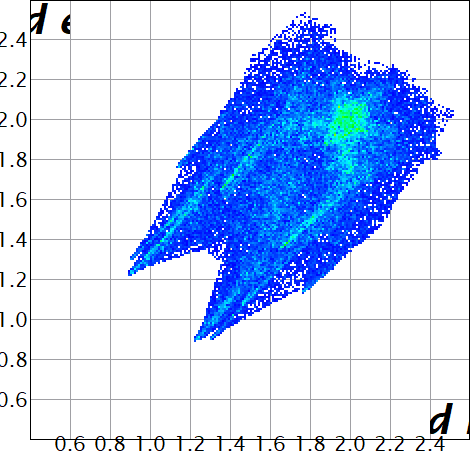


Figure 5. Triclinic Form I fingerprint.

1. **Interaction Energies.**


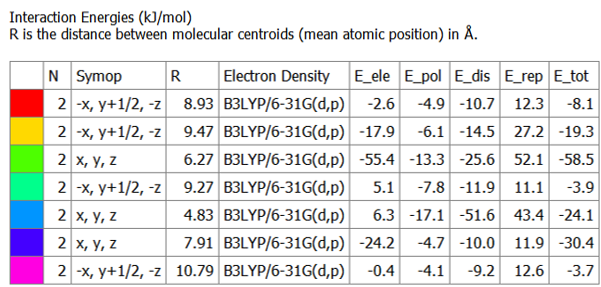


Table 1. Pairwise interaction energies for Monoclinic Form III.


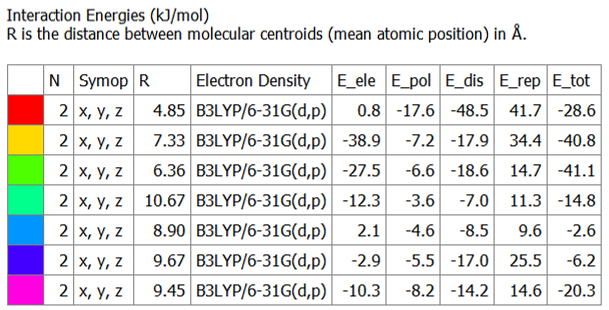


Table 2. Pairwise interaction energies for Triclinic Form I.
